# Handedness of a Motor Program in *C. elegans* Is Independent of Left-Right Body Asymmetry

**DOI:** 10.1371/journal.pone.0052138

**Published:** 2012-12-27

**Authors:** Joanna C. Downes, Bilge Birsoy, Kyle C. Chipman, Joel H. Rothman

**Affiliations:** Department of Molecular, Cellular and Developmental Biology and Neuroscience Research Institute, University of California Santa Barbara, Santa Barbara, California, United States of America; Harvard University, United States of America

## Abstract

Complex animals display bilaterally asymmetric motor behavior, or “motor handedness,” often revealed by preferential use of limbs on one side. For example, use of right limbs is dominant in a strong majority of humans. While the mechanisms that establish bilateral asymmetry in motor function are unknown in humans, they appear to be distinct from those for other handedness asymmetries, including bilateral visceral organ asymmetry, brain laterality, and ocular dominance. We report here that a simple, genetically homogeneous animal comprised of only ∼1000 somatic cells, the nematode *C. elegans*, also shows a distinct motor handedness preference: on a population basis, males show a pronounced right-hand turning bias during mating. The handedness bias persists through much of adult lifespan, suggesting that, as in more complex animals, it is an intrinsic trait of each individual, which can differ from the population mean. Our observations imply that the laterality of motor handedness preference in *C. elegans* is driven by epigenetic factors rather than by genetic variation. The preference for right-hand turns is also seen in animals with mirror-reversed anatomical handedness and is not attributable to stochastic asymmetric loss of male sensory rays that occurs by programmed cell death. As with *C. elegans*, we also observed a substantial handedness bias, though not necessarily the same preference in direction, in several gonochoristic *Caenorhabditis* species. These findings indicate that the independence of bilaterally asymmetric motor dominance from overall anatomical asymmetry, and a population-level tendency away from ambidexterity, occur even in simple invertebrates, suggesting that these may be common features of bilaterian metazoans.

## Introduction

Behavioral handedness asymmetry has been observed in many animal species. Humans are the most familiar example, with about 90% of the population favoring use of the right hand [1]. Chimpanzees also prefer one hand over the other, which leads to more successful foraging compared with non-lateralized (ambidextrous) individuals [2]. Left-right (L-R) behavioral asymmetry has also been observed in invertebrates, including turning direction in a maze with the giant water bug, *Belostoma flumineum* [3], laterally preferential eye use in the octopus, *Octopus vulgaris* [4], and asymmetric mating behavior in various species of hermaphroditic pond snails [5]. In the last case, the asymmetric behavior correlates with internal organ and brain asymmetry and shell chirality [6], and the behavioral handedness is likely to result simply from the geometric constraints of the body plan. In contrast, motor handedness in humans is not correlated with gross anatomical asymmetry. Individuals with reversed internal organ asymmetry (*situs inversus totalis*) show the same frequency of right-handedness as those with normal organ asymmetry [7]. Further, motor handedness in humans correlates only very weakly with asymmetry in brain function between right and left hemispheres [8].

While these examples are seen in complex animals with many cells, it is of interest to know whether motor handedness, and its distinction from anatomical handedness, is a characteristic of much simpler multicellular animals, and hence may be a pervasive characteristic of metazoans. The nematode *Caenorhabditis elegans*, a simple animal containing only ∼1000 cells, is a useful model organism with which to investigate L-R asymmetry. The profound L-R asymmetry in the arrangement of organs has been well-described in *C. elegans* [9]–[11]. This anatomical asymmetry is established in the early embryo at the 4- to 6-cell stage as a result of the skewed lateral division of two cells, called ABa and ABp, which leads to an embryo with defined (“dextral”) chirality. The ensuing intercellular signaling and developmental events result in profound L-R morphological differences [12]–[19] and some nervous system functional differences [20]–[23] in the adult. Worms with mirror-image L-R anatomical asymmetry arise from embryos in which the direction of skewing of the ABa/ABp division is reversed as a result of physical manipulation of mitotic spindles [10] or a mutation in the *gpa-16* gene [11]. Though the processes that establish anatomical asymmetry in *C. elegans* have been investigated [10], [11], no L-R asymmetric motor behavior similar to that seen in other animals has been reported in this creature, perhaps because it is generally cultured and studied on an agar surface, a predominantly two-dimensional environment that constrains movement along the L-R axis.

We report here that the turning behavior of male *C. elegans* worms during mating shows a pronounced bilateral asymmetry that persists through an extended period of adulthood. While the populations of both wild-type and several mutants show an overall preference for right turns, each population is significantly heterogeneous, generally with a small fraction strongly favoring left turns. Given the genetic homogeneity of *C. elegans*, the difference in handedness preference among individuals is best explained by epigenetic mechanisms. This proclivity towards a right-turning bias is also maintained in animals with reversed internal organ asymmetry, indicating that the symmetry-breaking event that establishes motor handedness bias is distinct from that responsible for creating embryonic chirality and L-R anatomical asymmetry. It is also independent of the L-R asymmetry in loss of male sensory rays that arises from stochastic apoptosis. Other *Caenorhabditis* species, both hermaphroditic and gonochoristic, also show a significant population-level heterogeneity and tendency away from ambidexterity, though the specific directional preference is not common to different members of the genus. Our results indicate that even a simple animal displays behavioral handedness asymmetry created by a symmetry-breaking mechanism that is distinct from that responsible for L-R anatomical handedness. This characteristic, along with a tendency away from ambidexterity in the population, is similar to that seen in much more complex animals, such as humans, and may therefore be a pervasive feature of metazoans.

## Results

### Identification of a Bilaterally Asymmetric Motor Trait in *C. elegans*

**Figure 1:**
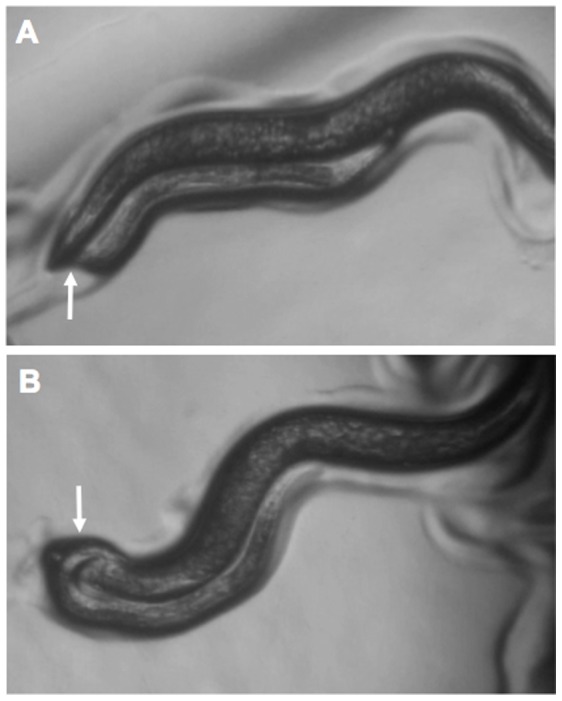
Left-right male turning behavior during mating. (**A**) Right turn (here, a difficult “under” turn). The male is lying on its right side and turning right, causing it to pass underneath the hermaphrodite. (**B**) Left turn (here, an easy “over” turn). The male is lying on its right side and turning towards its left to pass over the hermaphrodite. Arrows indicate points where the male’s tail passes over or under the hermaphrodite's body.

We sought to determine whether an animal with a simple body form exhibits motor handedness by examining several *C. elegans* movements for L-R asymmetry. While this animal moves on its right or left side primarily in two dimensions in contact with the surface of agar when cultured in the laboratory, some behaviors are performed in three-dimensions. We observed no striking L-R handedness bias for two such behaviors: nictation, the head-waving movement of the dispersal stage dauer larva [24], or pirouetting, the frequent turning associated with chemotaxis [25]. However, we found that mating *C. elegans* males do show a pronounced motor handedness bias. During mating, the male lies on its side and slides its ventral surface backwards along the dorsal or ventral surface of the hermaphrodite, searching for the vulva with its specialized tail structure [26]. When the male nears the hermaphrodite's head or tail, it makes a sharp body bend toward its ventral side, resulting in a 180^o^ turn around the dorsal-ventral axis of the hermaphrodite's body to continue this behavior on the opposite surface of its mate. We observed that a male generally performs each turn in three dimensions (Fig. 1A, B; Video S1), by moving towards either its left or right side as it makes the turn. Collectively, we found that as a population, males showed a clear handedness bias, or “mating handedness,” during repeated turns.

### The Wild-type *C. elegans* Population Shows an Overall Right-handed Bias

**Figure 2:**
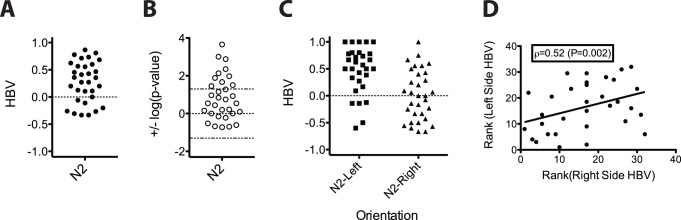
The wild-type (N2) *C. elegans* male population is predominantly right-handed. (**A**) HBV distribution of individual N2 worms (n = 32). (**B**) Plot of the log(p-value, two-tailed binomial test) for the data shown in (A). Right-handed worms are plotted with -1*log(p-value), whereas worms making predominantly left-handed turns are plotted with +1*log(p-value). Dashed lines represent log(p = 0.05). A majority of N2 worms scored were right-biased, and 10 were significant at the 0.05 threshold. (**C**) HBV for the N2 data partitioned based on orientation, which is demonstrably a significant factor in influencing turning behavior. (**D**) Plotting the ranks of the HBVs on the partitioned data reveals a significant correlation, indicating that individual biases in handedness are consistent across sides.

We quantified the proportion of right and left turns performed by young wild-type (N2) males during mating on an agar surface, allowing us to assess the handedness bias value (HBV; see Materials and Methods) of each individual. This analysis revealed a substantial right-hand bias in the male population (Fig. 2A). We observed no difference in handedness preference after animals were manipulated with a platinum pick, including when they were flipped from one side to the other during scoring; hence, the trait is robust to physical perturbation. Moreover, as males very often turned in the same direction in succession while mating with a hermaphrodite, there appears to be no strong influence of hermaphrodite dorsoventral orientation relative to the male.

To evaluate the significance of these data, we considered turning direction as a binary event in which the behavior of individual worms is assessed by a binomial test and the behavior of the entire sample of worms is modeled with a Generalized Linear Model (GLM; see Materials and Methods). Many N2 males (10/32) exhibited a significant (p<0.05, based on two-tailed binomial test) right-handed bias and none (0/32) showed a left-handed bias at the same significance threshold (Fig. 2B). Moreover, the probability of a right-handed turn (given hereafter as π) based on the GLM was 0.621, a highly significant difference (p = 8.2×10^−10^) from that expected for random turning (π = 0.5). While a large fraction of the males are right-handed, the population showed highly significant overdispersion (heterogeneity; p = 2.9×10^−8^; χ^2^ = 94.2; degrees of freedom = 31; see Materials and Methods), indicating that the population is, at a minimum, a mixture of right-biased and ambidextrous worms.

The turning behavior of a male is substantially affected by its L/R orientation on the surface of a culture plate owing to the resistance encountered when it turns under its mate toward the agar (“difficult” turns, Fig. 1A; compare to “easy” turns, Fig. 1B). Difficult turns are those made towards the agar and easy turns are those made away from the agar (where no resistance is encountered) irrespective of whether the male is making a left or a right turn. By separating the data for males oriented on their left *vs.* right sides (Fig. 2C), we found that males contacting the agar surface on their left side showed a very strong bias toward right (easy) turns, while those lying on their right side made easy turns about half the time (Fig. 2C). This effect of L-R orientation on the plate was highly significant (as assessed by including “Orientation” as a covariate in the GLM; p = 4.7×10^−11^). Nonetheless, the *relative* left- or right-handedness biases of the individual worms were significantly consistent across both orientations (based on comparison of p-values from a one-sided binomial test; Spearman’s rank correlation = 0.37; p = 0.017; Fig. 2D). Thus, while the male population shows a substantial right-hand bias, the impediment to turning caused by the agar is a constant factor that influences the behavior of individual worms equally. We observed similar results with other strains and species (see below): 4 of 5 *C. elegans* strains, and 3 of 4 other *Caenorhabditis* species, were significantly consistent in handedness preference between the two orientations (Table S1, Fig. S1), and the aggregate analysis of all 9 populations showed strong evidence for consistency (p = 1.23×10^−5^; see Table S1 legend).

### Persistence of Motor Handedness Bias through Adulthood

**Figure 3:**
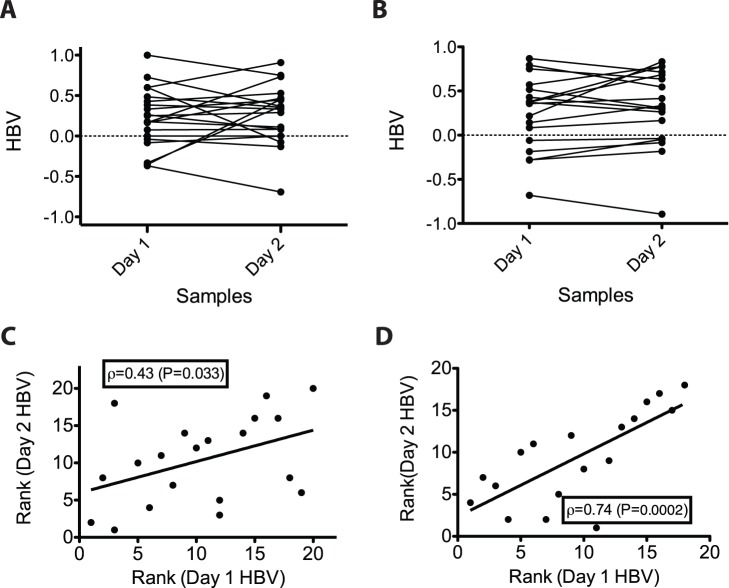
Persistence of handedness preference over two days of adulthood for N2. (**A**) and *gpa-16(-)* animals (**B**). Symbols on the left indicate Day 1 HBVs, symbols on the right indicate Day 2 HBVs for each worm scored. Plotting the rank of the HBVs reveals significant consistency in behavior over a two-day period for (**C**) N2 and (**D**) *gpa-16(-)* worms.

We next asked whether the motor handedness bias of individual worms is a persistent trait, as is the case with much more complex animals, by comparing the HBVs on the first and second day of adulthood, the period of peak mating efficiency [27]. We found that the HBVs of most males did not change dramatically over the two days, with a statistically significant consistency between Day 1 and 2 over the entire set analyzed (p = 0.018, based on Spearman's rank correlation on the p-values derived from a one-sided, “right-handed,” binomial test between the two days; Fig. 3A, C). Thus, males generally maintain a constant handedness preference, suggesting that this bias is an intrinsic and persistent attribute of each individual.

### Motor Handedness Bias is Independent of Anatomical Handedness

**Figure 4:**
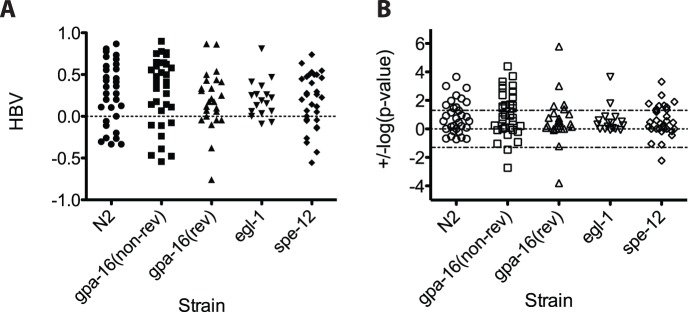
Motor handedness bias is independent of internal organ and ray asymmetry and hermaphrodite self-fertilization ability. (**A**) HBVs of individual worms for N2, non-reversed *gpa-16(-),* reversed *gpa-16(-)*, *egl-1(-)* and *spe-12(-)* samples. (**B**) Log (p-value two-tailed binomial test) of individual worms of the genotypes noted in (A), and plotted as described in Fig. 2. As with the N2 population, there is considerable heterogeneity among both normal and reversed *gpa-16(-)* animals according to the GLM model fit (non-reversed: p = 1.3×10^−7^; reversed: p = 8.4×10^−15^).

**Figure 5:**
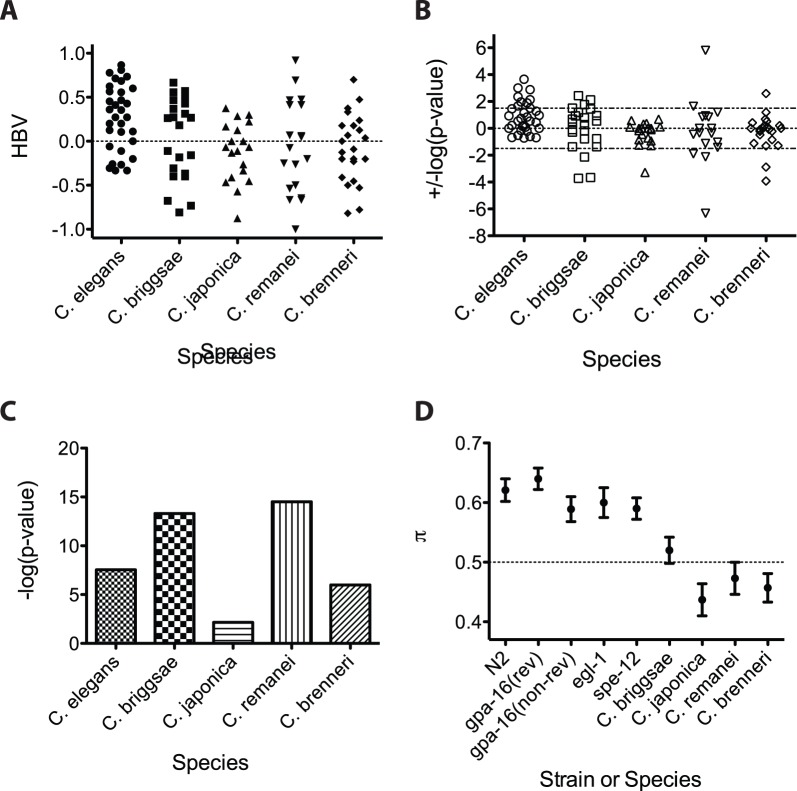
Motor handedness bias of five *Caenorhabditis* species. (**A**) HBVs of individual worms for N2 (n = 32), *C. briggsae* (n = 23), *C. japonica* (n = 19), *C. remanei* (n = 19) and *C. brenneri* (n = 23) samples. (**B**) Log (p-value of two-tailed binomial test) of the same worms as in (A) and plotted as in Fig. 4. (**C**) Log transformation of the p-values representing binomial logistic regression model fits for the indicated species. (**D**) Estimates of the binomial logistic regression π parameter for all 9 samples used in this study. There are 10 possible pairwise comparisons between the 5 N2-related strains; only one was marginally significantly different at the 0.05 threshold (*spe-12* vs. *gpa-16* reversed; p = 0.047). By contrast, all 5 N2-related strains are significantly different from each of the other 4 *Caenorhabditis* species (20 possible pairwise comparisons).

*C. elegans*, like many other animals, displays very pronounced internal organ asymmetry. In males, for example, the gonad is nearly always located on the right and the gut on the left of the body midline [10]. This largely invariant bilateral asymmetry arises during the embryonic developmental transition from a 4-cell embryo to a 6-cell embryo as the result of the skewing of L-R divisions of the ABa and ABp blastomeres along the anteroposterior axis, leading to a L-R asymmetry in the arrangement of cells in the embryo [9]. This skewing event is sufficient to create the overall bilateral asymmetry of the animal, as reversing the handedness of the divisions by physical manipulation [10] or mutation [11] results in adults with reversed anatomical chirality. To address whether mating handedness stems from this symmetry-breaking event in the embryo, we tested *gpa-16(it143)* mutants. ∼20% of adults produced by *gpa-16(it143)* mothers exhibit mirror-reversed L-R internal organ asymmetry owing to reversal in the early L/R skewing event [11], but appear otherwise phenotypically normal. Similar to wild-type males, *gpa-16(-)* males with normal L/R anatomy display a clear right-hand mating bias, showing that the *gpa-16* mutation *per se* does not substantially affect motor handedness (Fig. 4A, B; Fig. 5D; *π = *0.59, significantly greater than 0.5; p = 1.8×10^−5^). We found that complete reversal of the L-R body axis does not reverse motor handedness bias and, in fact, does not appreciably alter it: *gpa-16(-)* males with mirror-reversed anatomical symmetry also showed a highly significant bias toward right-handed turns (Fig. 4A, B; Fig. 5D; *π = *0.64, significantly above 0.5; p = 5.4×10^−10^). As with the wild-type population, the right-hand bias of *gpa-16(-)* animals persisted across two days (Day 1 and 2 were significantly consistent; p = 3×10^−4^; Fig. 3B, D). Pairwise comparisons between wild-type N2, non-reversed *gpa-16(-)*, and reversed *gpa-16(-)* strains showed no significant difference (p<0.05; when the covariate of “Strain” is incorporated into the GLM) in direction or magnitude in handedness bias. We conclude that the major symmetry-breaking system giving rise to L-R bias in motor handedness must be uncoupled from the event that generates the overall physical chirality of the embryo and organ asymmetry.

### Motor Handedness Bias is Independent of Asymmetry in Sensory Ray Number

*C. elegans* males use specialized sensory structures, or rays, embedded in the cuticular tail fan, to sense the hermaphrodite during mating [28]. While the male tail generally carries 9 bilateral pairs of rays, it is common for one or more rays to be missing in wild-type worms, producing a L-R asymmetry in the tail [28]. This loss in male rays is attributable to sporadic apoptosis and is eliminated by mutations in the *egl-1* gene (Choi et al, manuscript in preparation), which is essential for virtually all naturally occurring somatic cell apoptosis in the animal. Thus, virtually all *egl-1(-)* males contain a symmetric arrangement of rays. We found that, as in wild-type worms, *egl-1(n1084n3082)* males show an overall right-hand mating bias (*π = *0.60, significantly above 0.5; p = 6.2×10^−5^; Fig. 4A, B; Fig. 5D) that is not significantly different (p = 0.51) from that of wild-type N2 males. Thus, the natural, albeit sporadic, asymmetry in arrangement of male rays does not account for the L-R motor handedness of *C. elegans* males. Further, these results demonstrate that any bilateral asymmetry in the nervous system arising by asymmetric programmed cell death cannot be responsible for motor handedness asymmetry.

### Handedness Bias, but not its Direction, is a Common Characteristic of *Caenorhabditis* Species Irrespective of Gonochorism

If handedness in the male mating program is a positive attribute that improves mating efficiency, then one might expect it to be observed in related species. We examined mating handedness in other nematodes within the *Caenorhabditis* clade with the goal of asking 1) whether there is a tendency toward motor handedness bias in the mating behavior of other *Caenorhabditis* species and 2) if the direction of mating bias is evolutionarily conserved.

*C. briggsae*, like *C. elegans*, is a hermaphroditic nematode species that produces mating-competent males that are not essential. We found that *C. briggsae* males also turn left or right when they reach the termini of the hermaphrodite during mating. However, there was no strong bias toward either turning direction on aggregate at the population level (Fig. 5A, B, D; *π = *0.52, not significantly above 0.5; p = 0.36). Nonetheless, there were individual males that strongly favored either right or left turns. As with *C. elegans*, we observed highly significant heterogeneity (p = 4.8×10^−14^; Fig. 5C), suggesting that the *C. briggsae* population is likely to contain a mixture of left-handed, ambidextrous, and right-handed worms. Thus, while a tendency for favoring one turning direction in a given individual appears to be conserved, common to both *C. briggsae* and *C. elegans*, the strong right-hand bias of *C. elegans* is not.

If there is substantial selective pressure for mating handedness bias, such bias might be more pronounced in gonochoristic species compared to hermaphroditic species such as *C. elegans*, in which males are non-essential and arise only rarely owing to X chromosome nondisjunction. As an initial assessment of this question, we investigated the handedness of mating in the *C. elegans* mutant strain *spe-12(hc76); him-8(e1489)*, in which hermaphrodites are deficient in sperm production and cannot self-fertilize [29]. Propagation of this strain requires males to mate with otherwise sterile hermaphrodites; as such, the *spe-12(-)* animals are converted to gonochorism. This strain has been propagated in the laboratory for many generations, and thus male mating may have been subjected to selective pressure not experienced by wild-type *C. elegans*. However, we found that while the overall *spe-12(-)* population is right-biased (Fig. 4A, B; Fig. 5D; *π = *0.59; p = 1.22×10^−6^), this bias was no stronger than in wild-type N2 worms.

Next we turned to gonochoristic species, in which male mating has been under selective pressure for long periods. As with the hermaphroditic species, we found that males of the gonochoristic species *C. japonica* show a population-level tendency away from ambidexterity, but in this case with a significant left-ward bias (Fig. 5A, B; π = 0.437, significantly below 0.5, p = 0.023). Our results suggest that while *C. japonica* is a mixture of ambidextrous and left-handed worms (based on significant heterogeneity; p = 0.007; Fig. 5C), the species is not as strongly biased as might be expected if handedness of turning were under strong selection in this gonochoristic species. Further, these findings extend the observations with *C. briggsae* that the direction of handedness bias is not a character common to the *Caenorhabditis* clade.

A general population-level tendency away from ambidexterity in the *Caenorhabditis* clade was underscored by another gonochoristic species, *C. remanei*, in which more than half of the animals showed a strong bias towards either right or left turns (Fig. 5A, B). While there was little overall handedness preference across the population (π is 0.473; p = 0.32; Fig. 5D), this species showed highly significant heterogeneity (p = 3.1×10^−15^; Fig 5C), suggesting that left-handed, ambidextrous, and right-handed males are distributed across the *C. remanei* population.

Finally, *C. brenneri* showed weak evidence for a marginal left-handed bias in the population (π = 0.457, p = 0.07; Fig. 5A, B, D); however, the HBVs of two males were above, or close to, the 0.05 significance threshold, indicating that *C. brenneri* may also have some right-handed individuals. This population shows significant heterogeneity (p = 1.02×10^−6^; Fig 5C), suggesting that this species is a mixture of left-handed, ambidextrous, and right-handed animals.

We analyzed our findings to compare two quantitative parameters of L-R asymmetry in mating behavior: the mean of the HBVs, a measure of the overall left or right turning preference of the population (Fig. 5D), and heterogeneity, the tendency away from the mean as generated by the statistical model (Fig. 5C). All of the populations showed a weak-to-moderate mean handedness bias, indicating some degree of preference for left- or right-handed animals. Most of the populations showed strong heterogeneity, indicating that populations are a mixture of, at the least, two states of handedness. Indeed, of the nine populations in our study (5 N2 strains, 4 other species), we found, using the conservative Bonferroni correction (0.05/9 = 0.0056), that 5/9 samples were significant for population means deviating from 0.5 and 8/9 samples were significant for heterogeneity after correcting for multiple testing. This analysis also showed that heterogeneity and the strength of preference for a particular turning direction are separable: some species with strong lateral preference (high heterogeneity) show almost no bias toward one direction across the population, while others that show a strong directional preference are only weakly heterogeneous. Two populations in particular, *C. briggsae* and *C. remanei*, appear outwardly ambidextrous at the population level according to their mean HBV, but the high heterogeneity reveals that this is because each population contains a mixture of significantly right- and left-biased worms. Our findings suggest that a lateral preference in each individual of a species seems to be common across species, though the actual direction of bias may not be crucial.

## Discussion

In common with much more complex animals, including humans, we report that a simple creature with only ∼1000 cells can exhibit substantial L-R preference in the use of a motor program. We describe three major advances: 1) Males of a genetically homogeneous animal, *C. elegans*, show substantial motor handedness bias during mating that, as in humans, varies between individuals. The preference to make right or left turns is not affected by physical perturbation and is apparently retained throughout adulthood, indicating that it is a persistent characteristic intrinsic to each individual. While the population overall is right-biased, some worms show varying degrees of ambidexterity or left bias, the difference likely being determined epigenetically; this finding is consistent with the difficulty of identifying a genetic basis for motor handedness in humans. 2) The L-R handedness bias in the mating program is established by a mechanism that is independent of the symmetry-breaking mechanism that determines the unique anatomical handedness of the animal. Further, this L-R asymmetry is not the result of L-R asymmetric loss of sensory rays that are essential for the mating program. 3) The population-level tendency away from ambidexterity, though not the lateral direction of the bias, is prevalent in both hermaphroditic and gonochoristic species within the *Caenorhabditis* clade. These findings provide the opportunity to explore the genetic and evolutionary basis of population-based motor handedness.

The observations reported here address the extent to which motor handedness preference of any one individual is likely to be genetically determined. As propagated in the laboratory, all individuals in a culture of *C. elegans* are isogenic, i.e., virtually genetically identical [30]–[32]. Owing to self-fertilization, any genetic changes arising during propagation of a culture rapidly become homozygous. Thus, if the difference between right-preferring and non-right-preferring males is genetically determined, one would expect the distribution of these two types of individuals to fluctuate dramatically between cultures and strains. Yet we have observed a very similar distribution of lateral preference from experiment to experiment and among several different *C. elegans* strains that have been propagated separately for many generations (e.g., Fig. 5D). Moreover, while all of the *C. elegans* strains were remarkably consistent with each other, they were each statistically different from all of the related *Caenorhabditis* species (Fig. 5D; Fig. S2). (It is interesting to note, however, that they are similar to the other hermaphroditic strain, *C. briggsae*; Fig. 5D, S2). Thus, the variation in handedness preference between individuals is apparently determined by epigenetic factors arising during development, rather than genetic heterogeneity. If this is generally true for motor handedness in animals, it could explain why identification of a genetic basis for motor handedness preference in humans has been elusive [1].

Although left or right turning preference in any one individual *C. elegans* male from a genetically homogeneous population is apparently determined by epigenetic mechanisms, handedness of the male mating program in the species overall is nonetheless likely to be under genetic control. The variation seen between the various *Caenorhabditis* species confirm that species, and therefore presumably genetic differences, influence motor handedness preference. *C. elegans* provides a useful genetic system for analyzing motor handedness by, for example, the application of quantitative genetic approaches [33].

We found that, as in humans, motor handedness preference in *C. elegans* is strong, but not extreme: individuals with both left and right preferences are found in the population. Much like humans, *C. elegans* displays a high level of heterogeneity in the degree of bias among individuals. This contrasts with anatomical handedness, in which *situs inversus* is very rare in both the human and *C. elegans* populations. The overall population-level right-turning bias also contrasts with the stochasticity in L-R difference seen with pairs of olfactory neurons, in which the distribution of an olfactory neuron of a given specificity on the left *vs.* right is entirely random over the population [34]. The symmetry break that leads to motor handedness preference may be initiated by a stochastic process that is biased in one direction by other influences, much as appears to be the case for the symmetry break between equivalent pairs of cells in the embryo that become different by undergoing lateral cell-cell interactions [9].

Our findings reveal that, in addition to other processes [35], [36] that establish bilateral differences that do not occur with a particular handedness, there exist at least two independent systems that create handedness asymmetry in *C. elegans*: one in the early embryo that creates L-R organ asymmetry and one that results in laterality in a motor program, as described here (Fig. S3). While it may be somewhat surprising that internal organ asymmetry does not lead to motor handedness, this is consistent with the evolutionary variation in motor handedness that we have observed: related *Caenorhabitis* species with similar body plans vary substantially in motor handedness preference. It will be of interest to learn the mechanism of the latter symmetry break and when during development it occurs. In other studies (Choi et al., in preparation), we have found a L-R asymmetry in the frequency of stochastic loss of male rays. As we have shown here, this asymmetric loss *per se*, does not account for the L-R asymmetry in male mating behavior; however, it is conceivable that asymmetric ray loss and motor handedness arise by a single common mechanism which leads to both asymmetries. In any event, it is clear that neither the defined handedness of the nervous system, nor the difference in the number of body wall muscles on left and right [37], both of which arise from the defined chirality of the embryo, account for the motor handedness bias we have observed, since their reversal does not alter the behavioral asymmetry. Similarly, the asymmetry in mating behavior cannot result simply from the overall physical geometry of the animal, as appears to be the case in pond snails [6]. Future studies may reveal whether simple worms, like humans, use a variety of independent L-R symmetry breaking systems that result in different functions and structures, each with a defined handedness.

Our findings raise the possibility that the direction of turning during mating may be a selected trait. In particular, the general population-level tendency away from ambidexterity we observed in most species may provide a more efficient mating system, just as the reproducible chirality of organ geometry in many (but not all) nematodes ensures that organs pack together in a reliable fashion. However, gonochoristic species, in which selection for male mating efficiency is much stronger than in hermaphroditic species, did not show an increased proclivity towards either direction, suggesting that handedness bias may not be highly selected or even that selection might be in the direction of symmetric behavior. Another possibility is that the direction of turning is a learned trait supported by the reward of a successful turn, though we have no evidence for such a possibility. Finally, rather than selection for handedness *per se*, the L-R biases observed in each individual might simply reflect that a completely symmetric system is more difficult to achieve than one that is biased in one direction or the other.

## Materials and Methods

### General Maintenance of *C. elegans* and Strains Used

Worms were maintained as described by S. Brenner [38] at 20°C and were scored at room temperature. All strains were provided from laboratory stocks or from the Caenorhabditis Genetics Center. The following strains were used: N2 [wild type], JR3284 [*gpa-16(it143) I*], MT8735 [*egl-1(n1084n3082) V*], CB4951 [*spe-12(hc76) I; him-8(e1489) IV*], DF5081 [*C. japonica*], JU724 [*C. remanei*], CB5161 [*C. brenneri*], AF16 [*C. briggsae*], and CB1309 [*lin-2(e1309) X*].

### Generation of Male Worms

To develop male strains in the hermaphroditic species, 50–100 L4-stage hermaphrodites were picked into a solution of 7% ethanol in a microfuge tube and agitated at room temperature for about 1 h., followed by centrifugation at 2000 rpm for 1 min. The pelleted worms were plated onto a fresh NGM plate seeded with OP50 and incubated at 20°C. F1 male progeny were collected after several days and used to establish male stocks.

### Scoring Mating Turns

For single-day scoring, individual adult *C. elegans* male worms were picked onto plates containing 3–4 adult *lin-2(e1309)* vulvaless mutant hermaphrodites. When males attempt to mate with *lin-2* hermaphrodites, they cannot make contact with a vulva and thus spend more time turning around the hermaphrodite, allowing us to score more turns. Each male was left to recover from physical perturbation of transfer and then scored on both its left and right sides, with a minimum of 12, and an average of 20.7, turns scored per worm. We found that other *Caenorhabditis* species do not mate with *C. elegans lin-2* mutants, so males of each species were scored with hermaphrodites or females of the same species. Turns were characterized as “over” (above the surface of the plate) or “under” (toward the agar surface), and the side that the male contacted the agar was recorded to determine the left or right direction of each turn. The handedness bias value (HBV) for each male was calculated as HBV = (fraction right turns made –0.5)/0.5.

For multi-day scoring, individual L4 males were picked onto a plate containing 3–4 adult *lin-2* hermaphrodites and left at room temperature overnight. They were then scored as 1-day adults the next day and 2-day adults the following day in the same manner as the single-day scores.

### Selecting Males with Reversed Organ Asymmetry

For initial experiments, males were scored for normal or reversed handedness after mating was scored. Following matings, males were anesthetized with 5% levamisole, mounted on agar pads ventral surface up, and imaged at 40x or 63x magnification on a Zeiss AxioSkop 2 to determine the handedness of organ asymmetry. The gonad is positioned to the left of the gut in animals with reversed handedness, the opposite orientation of normal animals. In subsequent experiments, males were scored for organ asymmetry on a dissecting microscope directly on culture plates by using a platinum pick to orient the animals with the ventral side up.

### Statistical Analysis

We used Generalized Linear Models (GLMs) with a logit link function [39] (binary logistic regression) provided by the R programming environment to model the data on male turns. The binary response variable is modeled as a binomial random variable, which is the number of left/right turns for each worm. The general form is ln(π/1- π) = B_0_+ e. Therefore, the parameter π can be derived by fitting the intercept (B_0_) and solving for π. In specific cases, we incorporated in the GLM the covariates “Sample,” “Orientation,” or “Day,” in order to test for differences between Strains/Species, the side on which the male is contacting the agar when mating, and Days 1 and 2, respectively. The criterion of comparison between models is based on the residual deviance after including an additional covariate, along with the change in degrees of freedom. Significance of overdispersion (heterogeneity) was assessed with the chi-squared test on the null deviance (the fit of the null model). With the exception of the analysis based on orientation of males, all analysis was performed on the aggregate of data for all males regardless of orientation.

## Supporting Information

Figure S1 **HBVs by side (left columns) and rank of the HBVs on the partitioned data (right columns) for (A) the 5** ***C. elegans*** **strains and (B) the 4** ***Caenorhabditis*** **species analyzed in this study.**
(EPS)

Figure S2 **Heatmap displaying the -log(p-value) for all 36 pairwise comparisons between the 9 samples analyzed in this study.** All *C. elegans* strains are statistically similar to each other, and all are statistically different from the related *Caenorhabditis* species.
(EPS)

Figure S3 **Cartoon summarizing separate symmetry-breaking events in development.** The internal organ symmetry-breaking event occurs at the division of ABa and ABp, resulting in a L-R asymmetric body plan as shown in the cross section seen from the posterior end; the gut is on left and gonad on the right. The exact time at which the symmetry-breaking event responsible for motor handedness asymmetry occurs is unknown, but is likely prior to adulthood.
(EPS)

Table S1 **Summary of the consistency of behavior for individual worms across orientations (“Spearman's rho”).** The “Side Covariate p-value” is derived from the GLM, where we assess the significance of including the additional covariate. We found evidence of significant consistency in handedness between orientations for each of the populations except *egl-1* and *C. japonica*. The aggregate of all data for all 9 populations showed very strong consistency (Fisher’s combined probability test; p = 1.23×10–5).
(DOCX)

Video S1 **Video showing right and left turns during mating (same worm throughout video).** The worm is lying on its right side, requiring it to turn under its mate to make a right turn and over to make a left turn. The first half of the video shows right/under turns, and the second half of the video shows left/over turns. Videos were recorded with a digital camera mounted on the eyepiece tube.
(MOV)
