# Supplementary material for: Handedness of a Motor Program in C. elegans Is Independent of Left-Right Body Asymmetry
Source: PLoS One. 2012 Dec 27;7(12):e52138. doi: 10.1371/journal.pone.0052138 (PMC3531390; doi:10.1371/journal.pone.0052138)
Supplement: Table S1 — Summary of the consistency of behavior for individual worms across orientations (“Spearman's rho”). The “Side Covariate p-value” is derived from the GLM, where we assess the significance of including the additional covariate. We found evidence of significant consistency in handedness between orientations for each of the populations except egl-1 and C. japonica. The aggregate of all data for all 9 populations showed very strong consistency (Fisher’s combined probability test; p = 1.23×10^–5^). (DOCX) [file pone.0052138.s004.docx]

| Sample | n | Spearman's rho | Rho p-value | Side Covariate p-value |
| --- | --- | --- | --- | --- |
| N2 | 32 | 0.52 | 0.002 | 4.7E-11 |
| *spe-12* | 31 | 0.26 | 0.080 | 6.9E-07 |
| *gpa-16* reversed | 25 | 0.36 | 0.040 | 1.0E-05 |
| *gpa-16* non-reversed | 34 | 0.34 | 0.020 | 1.5E-10 |
| *egl-1* | 17 | -0.12 | 0.710 | 0.06 |
| C. brenneri | 23 | 0.40 | 0.030 | 9.9E-05 |
| C. remanei | 19 | 0.55 | 0.007 | 0.06 |
| C. japonica | 19 | 0.09 | 0.350 | 4.6E-10 |
| C. briggsae | 23 | 0.52 | 0.010 | 1.6E-06 |
